# Sex differences in the behavioral responses of dogs exposed to human chemosignals of fear and happiness

**DOI:** 10.1007/s10071-021-01473-9

**Published:** 2021-01-18

**Authors:** Biagio D’Aniello, Barbara Fierro, Anna Scandurra, Claudia Pinelli, Massimo Aria, Gün R. Semin

**Affiliations:** 1 Department of Biology, University of Naples Federico II, via Cinthia, 80126 Naples, Italy; 2 Department of Environmental, Biological and Pharmaceutical Sciences and Technologies, University of Campania “Luigi Vanvitelli”, Caserta, Italy; 3 Department of Economics and Statistics, University of Naples Federico II, via Cinthia, 80126 Naples, Italy; 4 William James Center for Research, ISPA-Instituto Universitario, 1149-041 Lisbon, Portugal; 5 Faculty of Social and Behavioral Sciences, Utrecht University, Utrecht, The Netherlands

**Keywords:** Dogs, Human chemosignals, Emotions, Sex differences, Animal cognition

## Abstract

This research focuses on sex differences in the behavioral patterns of dogs when they are exposed to human chemosignals (sweat) produced in happy and fear contexts. No age, breed or apparatus-directed behavior differences were found. However, when exposed to fear chemosignals, dogs’ behavior towards their owners, and their stress signals lasted longer when compared to being exposed to happiness as well as control chemosignals. In the happy odor condition, females, in contrast to males, displayed a significantly higher interest to the stranger compared to their owner. In the fear condition, dogs spent more time with their owner compared to the stranger. Behaviors directed towards the door, indicative of exit interest, had a longer duration in the fear condition than the other two conditions. Female dogs revealed a significantly longer door-directed behavior in the fear condition compared to the control condition. Overall the data shows that the effect of exposure to human emotional chemosignals is not sex dependent for behaviors related to the apparatus, the owner or the stress behaviors; however, in the happiness condition, females showed a stronger tendency to interact with the stranger.

## Introduction

In terms of their investment in the reproduction process, females are biologically preconfigured to nurture and care for their offspring; whereas, male fitness is marked by the number of females they have inseminated (Fitzpatrick et al. 1995; Rubenstein and Lovette 2009; Rosvall 2011). Such sex-specific differences of behavioral traits can emerge as a result of sexual selection via mate choice and intra-sexual competition (Schuett et al. 2010). Wolves (*Canis lupus lupus*), in the course of the domestication process, switched from natural (and sexual) selection to artificial selection. This change lowered the selective pressure on dogs (*Canis lupus familiaris*) for essential survival traits (Price 2002) but increased their socio-cognitive skills allowing them to adapt to the anthropogenic niche. Most of the success of dogs in our society relies on their increased social attention toward humans (Virányi et al. 2004; Mongillo et al. 2015; Alterisio et al. 2019). Dogs can adequately understand both our verbal and non-verbal stimuli (Mills 2005), hundreds of words (Kaminski et al. 2004; Pilley and Reid 2011) relying on specific neural correlates to process word meaning as well as intonation (Andics et al. 2016). Moreover, they are able to interpret and respond correctly human gestures (D’Aniello et al. 2016, 2017; Scandurra et al. 2017, 2018a; Grassmann et al. 2012). As recent research suggests, they can differentiate human emotions via chemosignals (Siniscalchi et al. 2016; D’Aniello et al. 2018; Semin et al. 2019) and by processing visual and acoustic signals (Turcsán et al. 2015; Siniscalchi et al. 2018a, b). Aside from being very skilled as cue decoders, dogs are also able to send messages, for example, by asking humans for help when they encounter some problem, using gaze behavior or physical contact (Miklósi et al. 2003; Scandurra et al. 2015; D’Aniello et al. 2015; D’Aniello and Scandurra 2016).

Despite the fact that the anthropogenic niche has reduced the impact of natural and sexual selection, artificial selection processes have not changed sex differences in several cognitive domains (Scandurra et al. 2018b). Consistent individual behavioral tendencies (personality traits) as a function of sex differences in dogs have been reported. Male dogs appear to express a higher degree of aggressiveness compared to female dogs (Borchelt 1983; Pérez-Guisado et al. 2006; Asp et al. 2015). Aggressiveness is linked to boldness in a specific aggression–boldness syndrome (Sih et al. 2004) and indeed, male dogs appeared to be bolder than female dogs in several experimental contexts (Svartberg 2002; Wilsson and Sundgren 1997; Asp et al. 2015). In contrast, female dogs appeared to be more sociable than male dogs, showing more friendly behaviors and making more physical contact with strangers (Lore and Eisenberg 1986; Wilsson and Sundgren 1997). There is evidence that the tendency for higher sociability observed in females may be genetically encoded (Persson et al. 2015). Nevertheless, in other types of social interaction contexts, such as interspecies play, male dogs showed more willingness to play with humans compared to female dogs (Strandberg et al. 2005; Asp et al. 2015). Sex differences in dogs were also found for other cognitive processes. Males appeared to be more flexible than females in changing their navigation strategies when forced to switch from their preferred (allocentric) to a non-preferred (egocentric) strategy (Topál et al. 2006). However, females appeared more skillful than males in learning a navigation task in a T maze (Mongillo et al. 2017). Lateralization was also reported to be sex dependent, with most studies reporting male dogs prevalently to be left-pawed, while females appeared prevalently right-pawed (Wells 2003; Quaranta et al. 2004). However, this effect might be weak since some studies were not able to replicate these results (Branson and Rogers 2006; Poyser et al 2006; Schneider et al. 2013). Regarding the perceptual level, females seem to rely more on the visual domain than males, both in social (Duranton et al. 2016; Mongillo et al. 2016; D’Aniello et al. 2016) and physical cognition contexts (Müller et al. 2011; Rooijakkers et al. 2009). Furthermore, in the case of olfactory discrimination, only male dogs appear able to discriminate kin (Hamilton and Vonk 2015). Males are also attracted by vaginal secretions more than females. In contrast, females appeared more persistent than males when investigating food odors (Siniscalchi et al. 2011). Although exploring sex differences in dogs is a flourishing research field, to our knowledge, research reporting differences in emotional reactivity by the two sexes are missing.

The current study extends our earlier research (D’Aniello et al. 2018) by examining sex differences in behavioral patterns of male and female dogs in response to human chemosignals (sweat) produced under happy and fear conditions. Communication via chemosignals is the most widely used form of communication among intraspecies. Indeed, even plants (Heil and Karban 2010) and bacteria (Taga and Bassler 2003) have been shown to rely on communication via chemosignals. The range of information that chemosignals carry is wide. Among the different types of information transmitted by chemosignals, emotional information has been examined extensively in humans (see Semin and de Groot 2013; de Groot et al. 2017). These studies have revealed that body odors (chemosignals) collected from an individual induces a simulacrum of the emotional state of the sender in a receiver—a type of synchrony driven by chemosignals (Semin 2007). Aside from intraspecies communication by means of chemosignals among humans, it has been shown that human emotions can be conveyed to other species, in particular dogs (Siniscalchi et al. 2016; D’Aniello et al. 2018) and horses (Lanatà et al. 2018; Sabiniewicz et al. 2020).

In our earlier research (D’Aniello et al. 2018), we used an experimental paradigm in which both an owner and a stranger were present in the room at the same time while the dogs were exposed to human sweat obtained in conditions of fear or happiness. The results revealed that dogs were more engaged in stranger-directed behaviors when they were exposed to happiness chemosignals. In contrast, human fear chemosignals induced more interest in owner-directed behaviors in the dogs, as well as a higher heartbeat rate and signaled more stress. These behavioral responses were indicative of a sort of empathetic emotional contagion mediated by chemosignals, inducing dogs to mirror the emotional status of the human sender (D’Aniello et al. 2018). The question we asked in this paper was whether there was a sex difference in the behavioral responses we had recorded in our earlier contribution (D’Aniello et al. 2018). This question was prompted by research showing that female dogs are less bold than males (Svartberg 2002; Wilsson and Sundgren 1997; Asp et al. 2015), which led us to expect that they would be likely to display higher, more frequent fear responses when exposed to fear inducing chemosignals. To examine this, we noted the duration with which female dogs displayed proximity to their owner, considered as their base of security (Prato-Previde et al. 2003). Aside from this, we observed the stress signals as well as attempts to escape the experimental room. We expected that female dogs would engage in these behaviors for a longer duration than male dogs. On the other hand, human females seem to be more skillful than males to perceive happy emotions, a property emerging very early during development (Rosen et al. 1992). If this sex difference also holds for dogs and affects their behavioral responses, considering that the human body odors of happiness trigger longer interactions with the stranger (D’Aniello et al. 2018), then a higher tendency to interact with the stranger would be expected for female dogs.

## Materials and methods

### Odor collection

The odors for our study were donated by 8 Caucasian—heterosexual males, 21 years old on average—students of the ISPA University in Lisbon (Portugal). After giving their informed consent, the donors participated on a voluntary basis in two sweat collection sessions (fear- and happiness-inducing sessions), which were separated by a week’s interval. Participants were heterosexual, nonsmokers, not under any medication at the time of the collection and did not have any reported psychological or neurological disorders. Following previous guidelines regarding sweat collection (e.g., de Groot et al. 2017), only males were included as sweat donors because of their larger and more active apocrine glands than females (Zhou and Chen, 2009). The donors’ sweat was collected using specific sterile absorbent pads (Cutisorb, BSN Medical, Hamburg, Germany) under both the armpits of each donor. Emotions were elicited after watching 25-min videos inducing fear or happiness emotions separated by one week in the same person. After being removed, all the pads were stored at a temperature of − 22 °C and then sent to the Italian laboratory for the testing procedure on dogs. Here, the pads were cut into four pieces, each mixed with those of three different individuals to create a super sample, thus limiting the effect of interindividual differences in body odors (Mitro et al. 2012). All the procedures for the sweat collection were approved by the host institution ethics committee (Protocol Nr. 2017/0025509) and were conducted in accordance with the standards of the American Psychological Association and the guidelines of the Declaration of Helsinki.

### Subjects

Dog/owner dyads (35 Golden Retrievers, GR, and 76 Labrador Retrieves, LR) were recruited through personal contacts, advertisements in public places, veterinary surgeons and through the Internet. Some dyads had to be excluded because of fear-related problems manifested by the dogs (3 subjects) before starting with the testing session and were not admitted to the testing procedure. Some testing sessions were interrupted abruptly because the dogs displayed destructive behaviors towards the apparatus (*n* = 10) or because the owners did not comply with the instructions (e.g., interacting with either the dog or the stranger during the test) (*n* = 14). Overall, 27 dogs had to be excluded, while 84 remained with the following distribution: 28 dogs were in the happiness condition (14 males, 9LR + 5GR, age 41.5 ± 28.6 months, and 14 females, 9LR + 5GR, age 43.8 ± 38.9 months); 30 dogs were in the fear condition (15 males, 8LR + 7GR, age 40.5 ± 22.1 months, and 15 females, 12LR + 3GR, age 39.9 ± 25.9 months); 26 dogs were in the control condition (13 males, 9LR + 4GR, age 42.5 ± 29.1 months, and 13 females, 10LR + 3GR, age 27.8 ± 15.9 months). All the dogs resided in a family home and had close contact with people. However, the housing condition of the dogs was unknown. About 20% of the neutered dogs were distributed equally across the conditions. Thus, the database for the current study included the additional recruitment of 44 dogs (along with the 40 dogs from the original study, D’Aniello et al. 2018).

### Apparatus and procedure

**Fig. 1:**
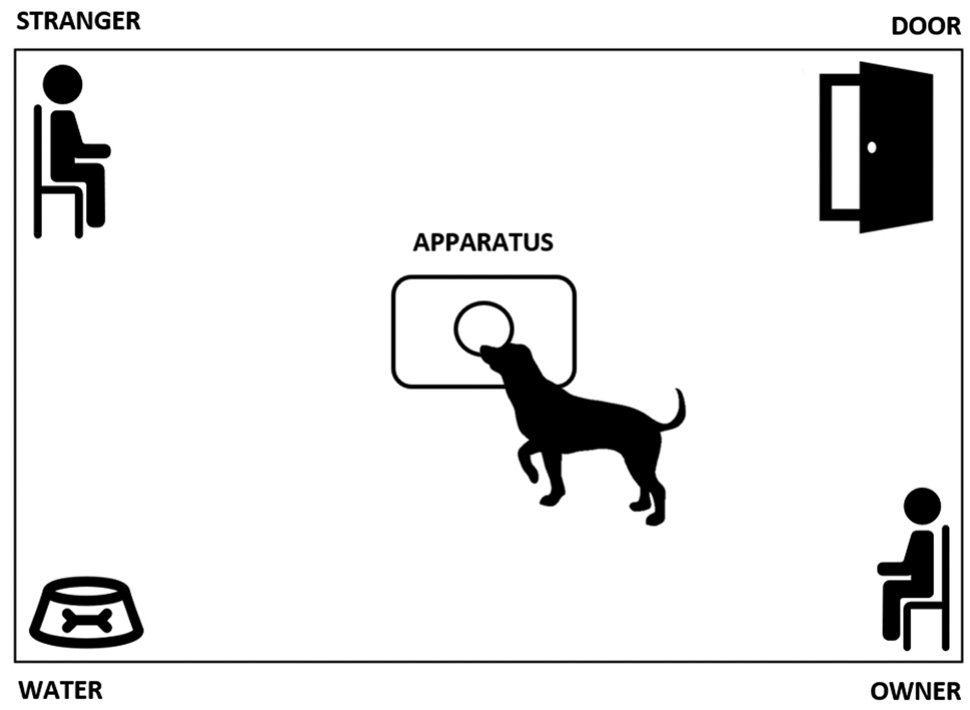
Graphical representation of the procedure room used for the tests. Two chairs at opposite corner hosted the dog’ owner and the stranger; a water’ bowl was placed in the corner opposite to the door exit; the apparatus containing the sweat sample was positioned in the center of the room

The tests were conducted at the University Federico II in Naples, in a procedure room (3.7 × 2.9 m) unknown to the dogs. The room temperature was set to 24 °C and was the same across the experimental conditions. After their arrival, the dog/owner dyads were welcomed by the laboratory staff before entering the room for a period of about 5–10 min, during which the dogs were allowed to drink ad libitum. Contact with the dogs was limited while the staff explained the procedure to the owner. Then, the dog and its owner entered the room, where an experimenter (E1), unknown to dog, was seated on a chair. The owner and E1 were seated in the two opposite corners of the room. A small bowl with water was placed in the corner of the room, opposite to the door exit (Fig. 1). In the meantime, a second experimenter (E2) baited the experimental apparatus with one of the three conditions chosen for the test: i.e. fear, happiness or control (unused pads). Neither the owners nor E1 took an active part in the test, and both were blind to the condition. The apparatus included a wooden base (39.5 × 30 cm) and a plastic container screwed to the base. The lid was provided with a hole (3 cm diameter) to allow dogs to sniff the odors. After 1 min of familiarization inside the room, the owner was asked to hold the dog close to the chair, to allow E2 to enter the room and set the apparatus at the center. As E2 left the room, the owner was asked to release the dog. The testing procedure lasted 2 min.

Both the E1 and owner remained in the room during the entire procedure and were instructed not to interact with the dog (they were given two magazines to avoid eye contact with the dog) during the test (even when solicited by the dog). Neither E1 nor the owner was aware of the condition (blind procedure). At the end of each test, the bowl, the apparatus and the room were washed and cleaned. To avoid possible contamination, three different but identical appearing apparatuses (one for each condition) were used. At the end of each test, the samples were frozen again and were reused for no more than 4 times. All the tests were recorded through a closed-circuit television system with 4 cameras located at the corners of the room.

### Behavioral parameters

We recorded the duration of all behaviors related to the apparatus, the door or the people (i.e., approaching, interacting and gazing) and categorized as apparatus, door-, owner- and stranger-directed behaviors. Stress behaviors were also pooled (Table 1). When two or more stress behaviors co-occurred, we recorded the one that lasted longer. The duration of each behavior was recorded using Solomon Coder^®^ beta 16.06.26 (ELTE TTK, Hungary). The data were coded by an expert researcher, while a second independent researcher randomly coded 16 videos (19%) of the total sample for to test for interobserver reliability. The level of agreement ranged by 93–99% depending on the ethological categories examined.

**Table 1:** Ethogram adopted during the test. We recorded the duration of behaviors denoting interest toward the apparatus, the door, the owner and the stranger and categorized them as target-directed behaviors including gazing, interaction and approach. The stress behaviors were also recorded

Behaviors	Description
Approach	The dog approaches the target. This behaviour was recorded when the dog was moving toward the door, the apparatus and the people (irrespectively whether it was gazing toward the face of other part of the body)
Interaction	The dog engages in physical contact with the target. It includes explorative behaviors, such as sniffing (from not more than 20 cm about). Furthermore, physical interaction with muzzle or legs, licking, jumping up the target were also included
Gazing	The dog looks at the target from a stationary position. Gazing behavior toward the people was recorded when clearly directed to the face of the subjects
Stress	All behaviors indicating a stressful response. Includes mouth licking (the dog licks its lips or nose), locomotion (dog walking, pacing or running around without a clear target or exploratory intent), shaking off, scratching, yawning, barking, yapping, panting, drinking water

### Data analysis

Most of the data were not normally distributed, as the Shapiro–Wilk test revealed; a re-scaling process by logarithmic normalization, as well as other procedures, was not effective for the datasets. Therefore, we opted for the Kruskal–Wallis non-parametric statistical test, followed by Mann–Whitney pairwise post hoc tests with Bonferroni correction. These tests were first applied to compare responses in the conditions independent of sex to obtain a general pattern and allow a comparison with our previous findings (D’Aniello et al. 2018). To study the contribution of sex in each condition, the data were grouped by sex and conditions resulting in six independent groups: females in happiness (F-happiness), fear (F-fear) and control (F-control) conditions; and males in happiness (M-happiness), fear (M-fear) and control (M-control) conditions. Furthermore, a Wilcoxon test was used as a test for comparing the interest between owner and stranger in each sex and condition. P-value was adjusted according to repeated measures. All statistical analyses were performed by Past software (2002).

## Results

No age differences were observed across the six groups (i.e., condition (3) by sex (2)) (Kruskal–Wallis test, *χ*^2^ = 2.98, *p* = 0.7). The statistic on the type of breed in each group was not possible due to the limited sample size. Globally, no breed differences were recorded comparing LR and GR in the variables examined (owner-directed behaviors: U = 723, *p* = 0.3; stranger-directed behaviors: U = 732.5, *p* = 0.8; apparatus-directed behaviors: U = 660, *p* = 0.9; stress behaviors: U = 685, *p* = 0.5; door-directed behaviors: U = 592.5, *p* = 0.1). No statistical differences were found among the medians of apparatus-directed behavior. However, the other behaviors were found to be affected by the variable conditions as well as the interactions between conditions and sex, as detailed below.

### Owner-directed behaviors

The general pattern for the duration of the owner-directed behaviors was found to be significantly different as a function of condition (*χ*^2^ = 20.59, *p* < 0.001). Post hoc tests revealed that fear triggered higher owner-directed behaviors than both happiness (U = 146.5, *p* < 0.001) and control (U = 180.5, *p* = 0.002), with no statistical difference between happiness and control.

When considering sex separately across the conditions, the statistical analysis of the behaviors in the six groups appeared again to be significantly different (*χ*^2^ = 20.59, *p* < 0.001). Post hoc tests showed significant differences between F-fear and F-happiness (U = 35.5; *p* = 0.039) with the latter showing a lower median value. M-fear reached a higher median value than M-happiness (U = 35.5; *p* = 0.031) and M-control (U = 180.5; *p* = 0.002). A significant difference was also observed between the response of M-fear and F-happiness groups (U = 29.0; *p* = 0.015).

**Fig. 2:**
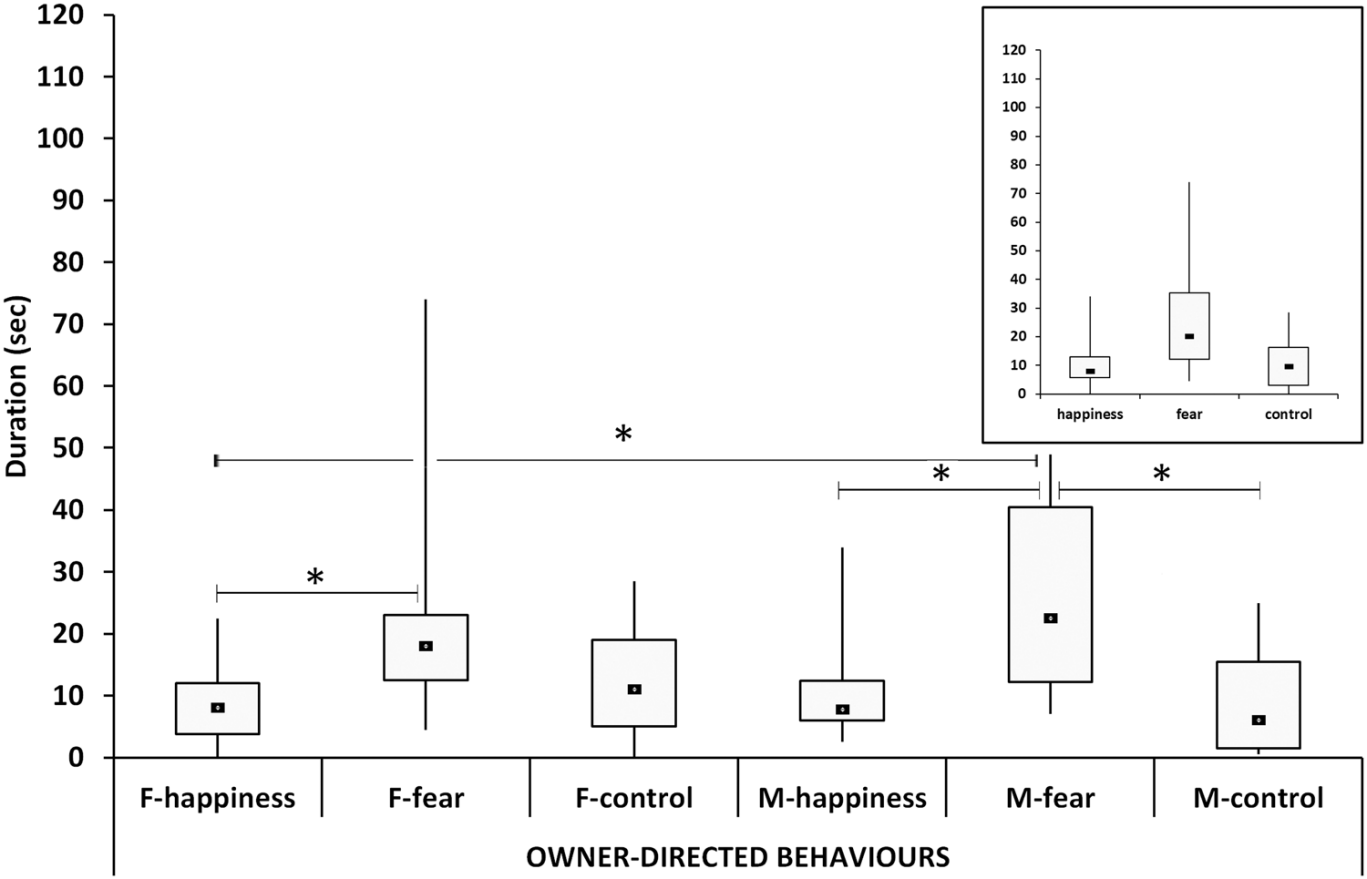
The duration of owner-directed behaviors. Black squares: medians; boxes: quartiles; thin vertical lines: minimum and maximum values. Horizontal lines with asterisks indicate significant post hoc differences. F-happiness = females in the happiness condition; F-fear = females in the fear condition; F-control = females in the control condition; M-happiness = males in the happiness condition; M-fear = males in the fear condition; M-control = males in the control condition. Patterns of males and females mirror the general pattern showed in the top right insert

Overall the patterns of males and females appeared to be very similar and both mirrored the general pattern with sex aggregates in the three conditions (Fig. 2).

### Stranger-directed behaviors

The overall pattern of the stranger-directed behaviors in the different odor conditions showed a different pattern of duration (*χ*^2^ = 16.31, *p* < 0.001), with happiness higher than both fear (U = 244.5, *p* = 0.019) and control (U = 141.5, *p* < 0.001) conditions and no differences between fear and control.

A significant different pattern emerged when comparing males and females separately across the odor conditions (*χ*^2^ = 21.50, *p* < 0.001). The median in the F-happiness group was significantly higher than all other groups (i.e. F-happiness vs. F-fear, U = 36, *p* = 0.025; F-happiness vs. F-control, U = 22.0, *p* = 0.013; F-happiness vs. M-happiness, U = 32.5, *p* = 0.042; F-happiness vs. M-Fear, U = 36.0, *p* = 0.041; F-happiness vs. M-control U = 15.0, *p* = 0.004). All other post hoc differences were not significant.

**Fig. 3:**
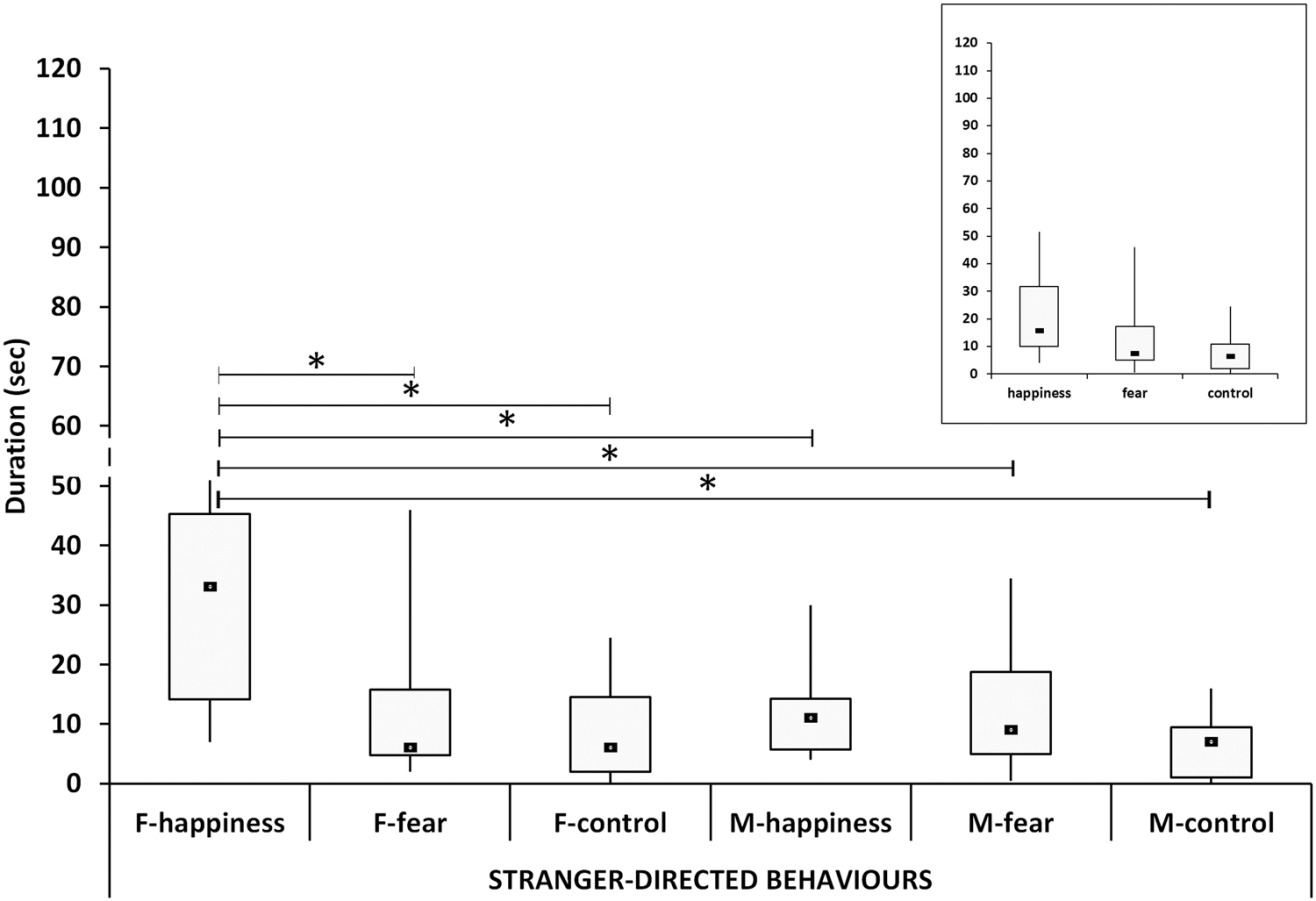
The duration of stranger-directed behaviors. Black squares: medians; boxes: quartiles; thin vertical lines: minimum and maximum values. Horizontal lines with asterisks indicate significant post hoc differences. F- happiness = females in the happiness condition; F-fear = females in the fear condition; F-control = females in the control condition; M-happiness = males in the happiness condition; M-fear = males in the fear condition; M-control = males in the control condition. Note that the patterns of males and females are different, with females mirroring more closely the general pattern showed in the top right insert

The patterns for males and females appeared to be very different, with females mirroring the general pattern more closely (Fig. 3).

### Comparative tests owner/stranger

The comparison between owner- and stranger-directed behaviors revealed that in the control condition, there was no statistical difference in dogs as a group, while the median in the fear condition was significantly higher for owner-directed behaviors (W = 390, *p* = 0.003). In contrast, the median of stranger-directed behaviors was higher in the happiness condition (W = 327, *p* = 0.015).

**Fig. 4:**
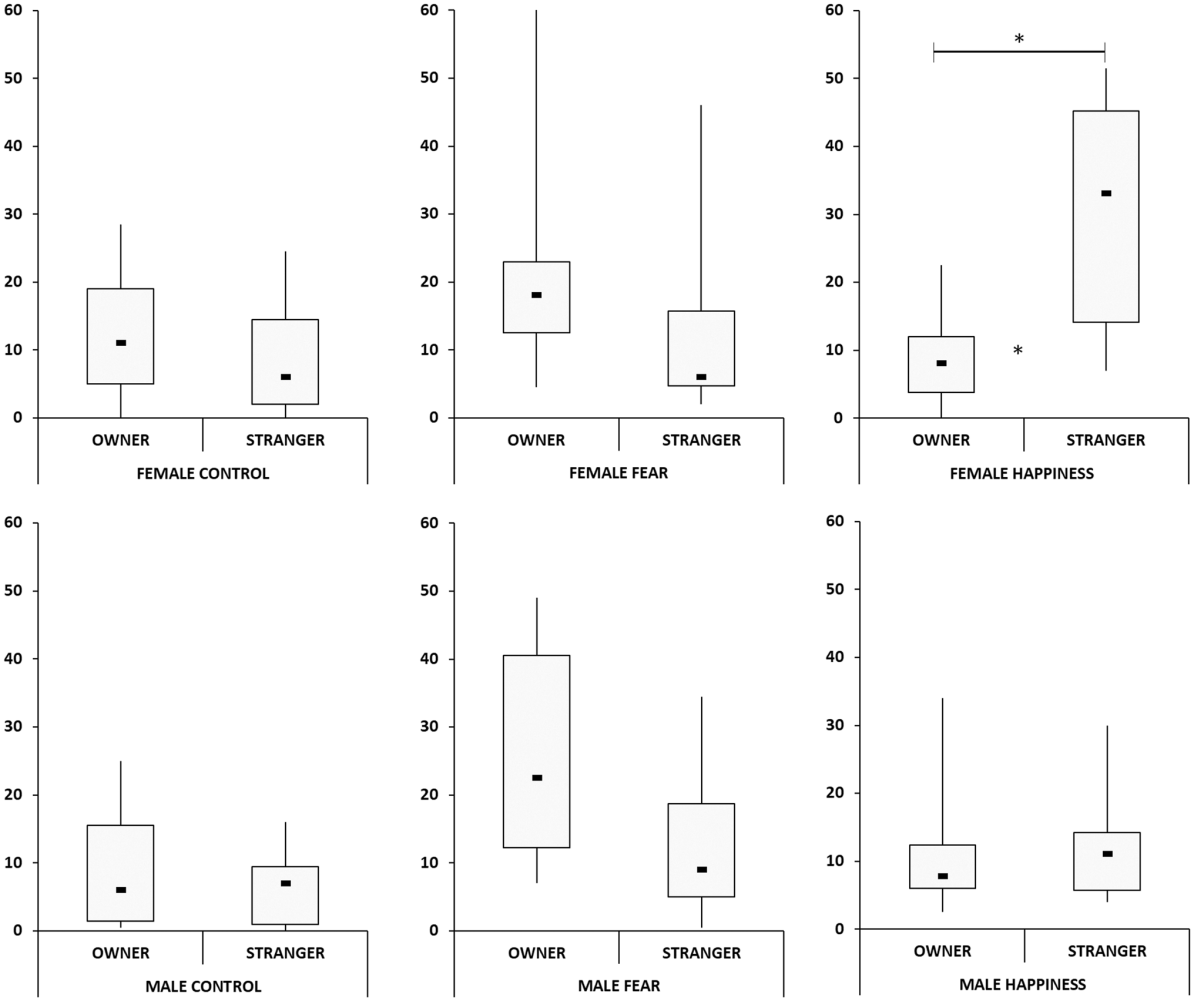
The duration of owner and stranger behavior in a comparative view. Left graphs showing no differences in the interest between the owner or the stranger in the control condition. Graphs on the center showing a significant interest for the owner in the fear condition in both sexes. Right graphs showing a significant interest for the stranger in females, but not in males in the happiness condition. Horizontal lines with asterisks indicate significant differences

Females clearly preferred the stranger over the owner in the happiness condition (W = 99.0, *p* = 0.024), whereas males showed no such tendency. Both males and females showed no differences in both the fear condition and control condition (Fig. 4).

### Stress behaviors

A significant difference was found for the stress behaviors among the conditions (*χ*^2^ = 32.5, *p* < 0.001). Post hoc tests revealed a higher median value in the fear condition compared to both the happiness (U = 200.0, *p* = 0.002) and control conditions (U = 63.5, *p* < 0.001). The median in the happiness condition was also found to be significantly higher than the control (U = 200.0, *p* = 0.014).

Analyzing sex separately yielded a significant difference between the groups (*χ*^2^ = 33.11, *p* < 0.001). Post hoc test showed stress behavior to be higher in the F-fear group compared to F-control (U = 15.5, *p* = 0.003) and M-control (U = 18.5, *p* = 0.005). The M-fear group revealed more stress signals than both F-control (U = 14.5, *p* = 0.002) and M-control (76.0, *p* = 0.002).

**Fig. 5:**
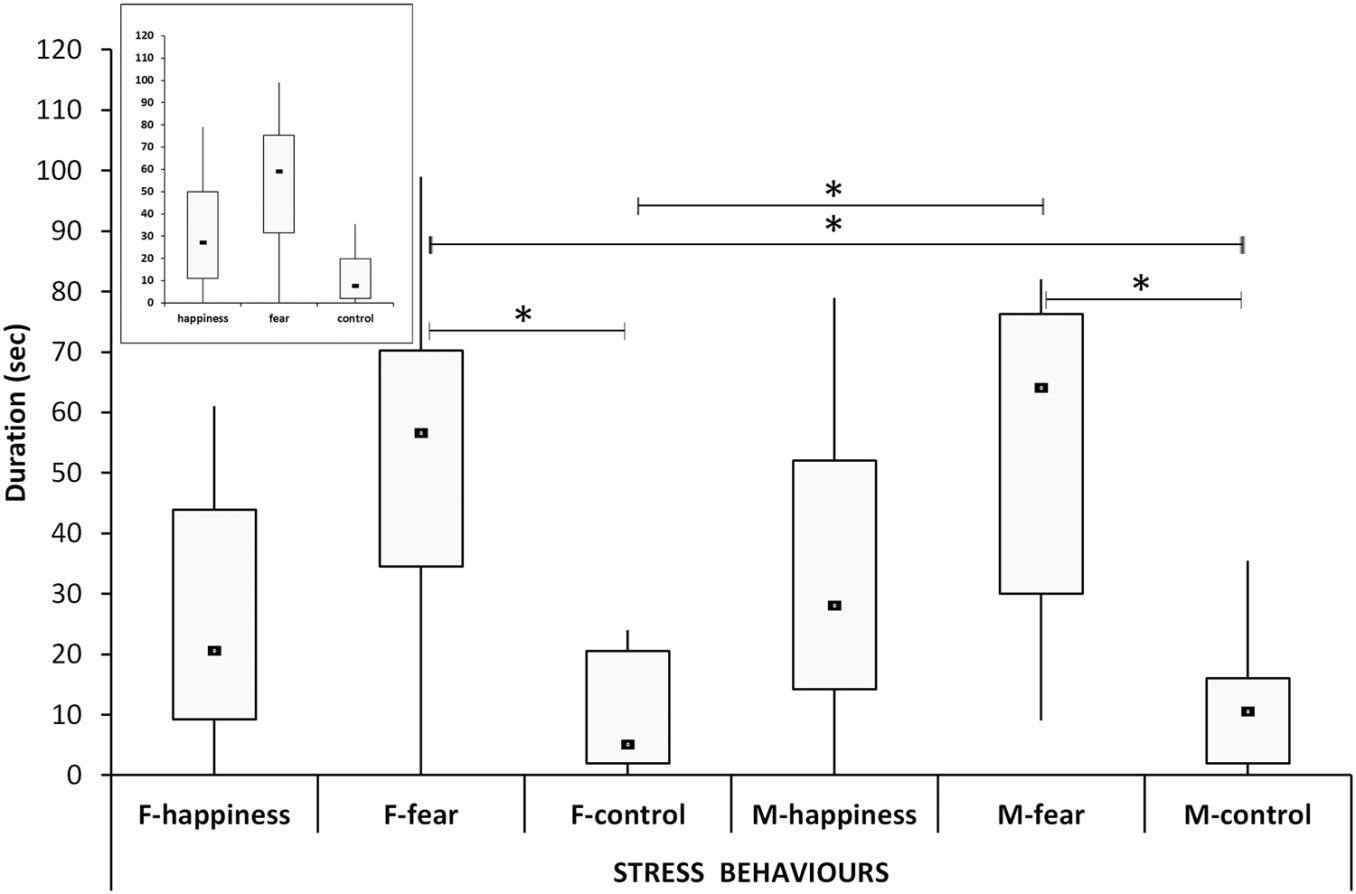
The duration of stress behaviors. Black squares: medians; boxes: quartiles; thin vertical lines: minimum and maximum values. Horizontal lines with asterisks indicate significant post hoc differences. F- happiness = females in the happiness condition; F-fear = females in the fear condition; F-control = females in the control condition; M-happiness = males in the happiness condition; M-fear = males in the fear condition; M-control = males in the control condition. Note that the patterns of males and females are similar and mirror the general pattern showed in the top left insert

Both females’ and males’ trends were similar and reflected very closely the general trend (Fig. 5).

### Door-directed behaviors

A significant difference was found between conditions in the door-directed behaviors (*χ*^2^ = 8.9, *p* = 0.012). Post hoc tests showed a higher median in the fear condition compared to both the happiness (U = 256.0, *p* = 0.034) and control conditions (U = 236.0, *p* = 0.035).

**Fig. 6:**
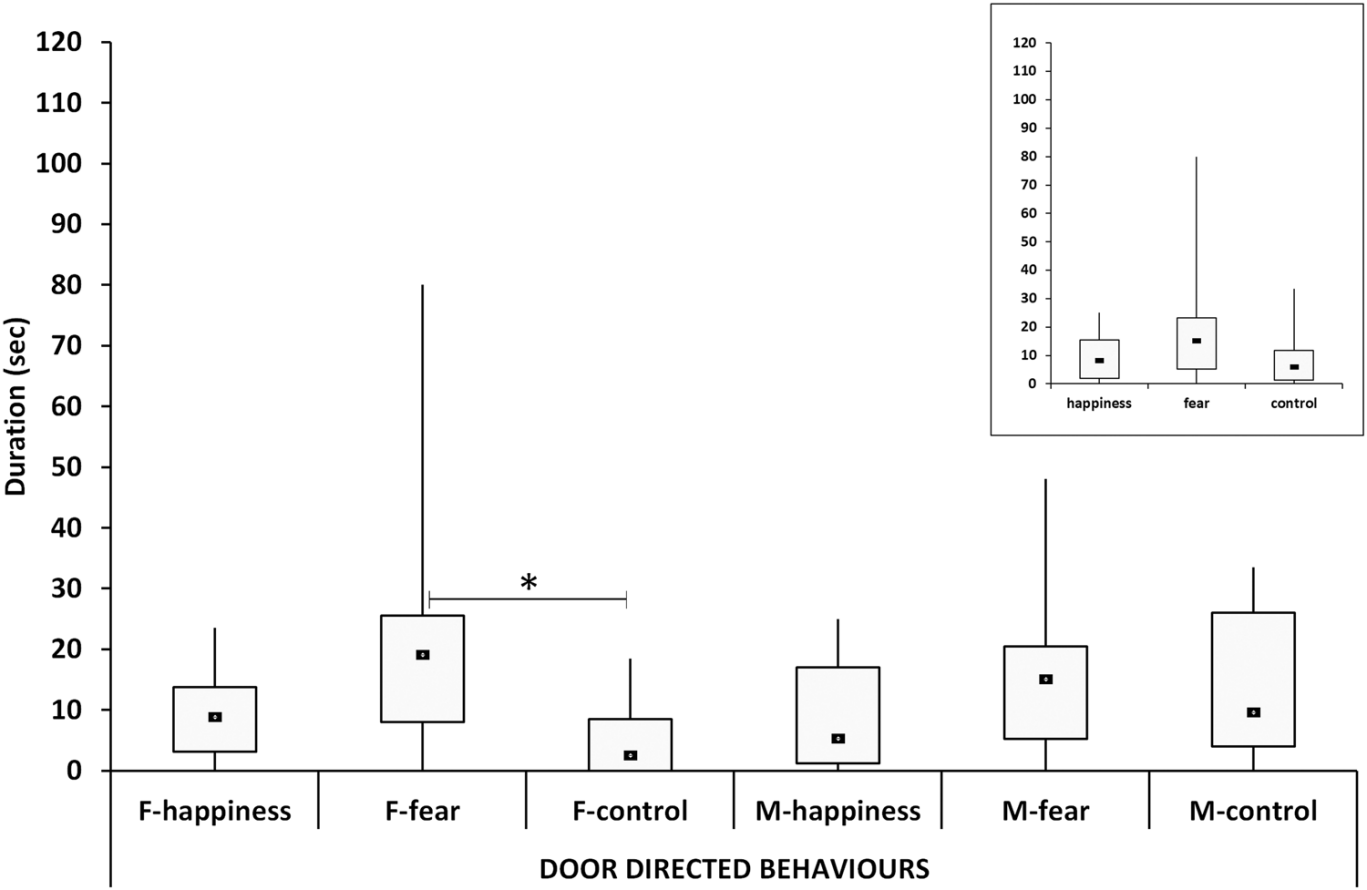
The duration of door-directed behaviors. Black squares: medians; boxes: quartiles; thin vertical lines: minimum and maximum values. Horizontal lines with asterisks indicate significant post hoc differences. F-happiness = females in the happiness condition; F-fear = females in the fear condition; F-control = females in the control condition; M-happiness = males in the happiness condition; M-fear = males in the fear condition; M-control = males in the control condition. Note that the patterns of females and males are quite similar, but females pattern appears more similar to the general trend showed in the top right insert

Considering the six groups, a significant difference was recorded (*χ*^2^ = 13.4, *p* = 0.019). A post hoc test showed a significantly higher median value in the F-fear group compared to F-control (U = 33.0, *p* = 0.047). The patterns related to females and males were quite similar, but females’ pattern appeared more similar to the general trend (Fig. 6).

## Discussion

Our results indicate that both males and females revealed similar patterns regarding owner-directed behaviors and the stress signals. They showed significantly more frequent owner-directed behaviors in the fear condition, but no sex difference effects. Both males and females displayed similar coping strategies choosing to refer to their owner and showing increased stress signals when exposed to human fear's chemosignals. These findings do not support our hypothesis that females would display a more fearful response due to lower boldness (Svartberg 2002; Wilsson and Sundgren 1997; Asp et al. 2015). However, females displayed a higher frequency of door-directed behavior in the fear condition compared to the control condition, while males did not differ in this behavior across the conditions. Thus, the door-directed behaviors supported our hypothesis predicting higher fearful responses from females. Similar behavioral patterns were observed for female mice (Archer 1975) and in women (Deng et al. 2016) in other experimental paradigms, where fear signals triggered a strong avoidance behavior compared to males.

In contrast to owner-directed behaviors, behavioral responses toward the stranger were clearly sex dependent. Indeed, while males showed no difference of stranger-directed behavior in the odor conditions, females showed higher stranger-directed behaviors in the happiness condition compared to the fear or control conditions. Moreover, the females in the happiness condition showed a clear interest for the stranger over the owner, a behavior not observed in male dogs. In a study based on male and female human-reaction tests, it was shown that females were much more likely to approach and make body contact with a stranger (Lore and Eisenberg 1986). Females appeared also more likely to interact with a stranger in the impossible task paradigm (Persson et al. 2015). Although these studies state a major tendency of females relating to unknown people, they do not describe the emotional condition of dogs and, hence, are not directly comparable with our data. Our result with the dogs is the first report of sex-dependent emotional responsiveness of happiness information carried by chemosignals.

In contrast to the fear condition, in which the dogs preferentially approached the owner, the happiness condition showed precisely the reverse. Increased stranger contacts may have resulted due to the happiness chemosignals inducing a more relaxed state in the dogs. In humans, happiness predisposes people to engage in contacting others (Baumeister and Leary 1995) as well as encouraging ongoing social contact (Hatfield et al. 1994). If this effect holds also for dogs, then it is possible that when experiencing a happy emotional state, dogs maintain more frequent social contact with strangers when exposed to happy human odors.

At the same time, this study underlines the robustness of the findings we reported earlier. The overall pattern of the data largely replicated the results obtained in our previous study (D’Aniello et al. 2018). Indeed, dogs as a group showed more owner-directed behaviors and stress signals in the fear condition, higher stranger-directed behaviors in the happiness condition with no differences in the apparatus-directed behaviors. Looking for the owner in unknown places has been considered a worrying behavior since dogs activate the attachment system, as shown in the strange situation test (Prato-Previde et al. 2003; Scandurra et al. 2016). This interpretation is confirmed by the same pattern shown by the higher stress signals recorded in the fear condition compared to the other conditions. In addition, we also found that dogs were more interested in the door in the fear condition compared to both the happiness and control conditions, with no difference between the happiness and control conditions. The door-directed behaviors could be indicative of an attempt to escape the room after being specifically exposed to emotional chemosignals of human fear. In the present study, we recorded a higher response in owner-directed behaviors when the dogs were exposed to the happiness odor compared to the control condition. Probably the general arousal caused by the chemosignals could explain this effect, something that we did not note in the previous study (D’Aniello et al. 2018). This may have been due to the smaller sample size in our previous study. Altogether, these findings indicate that human fear chemosignals have induced a comparable emotional state in dogs.

An interesting question is why males and females show different response patterns. From a specific evolutionary perspective, males and females are often involved in different roles within the social group (see for example Cassidy et al. 2017 for canids studies). Female dogs are more involved in parental care, where a better response to happiness could be advantageous in enhancing social relationships. This effect, in turn, improves the tolerance towards puppies and social relations in general, thus facilitating social cooperation in the care of offspring. While it is assumed that happiness does not carry as much “evolutionary salience” as fear (Zhou and Chen 2009), in women, the most important cause of happiness has been argued to be the presence of strong social relationships (Argyle 2001; Diener and Seligman 2002).

It should be underlined that the missing statistical response to stranger and door-directed behaviors in males do not exclude that they could have been affected by chemosignals to the same extent as females. We are not able to disentangle whether males and females differ at the sensory level, namely whether females experience more emotional responses or males have more controlled behavior after smelling human chemosignals. In humans, men appeared to show a similar sensorial perception of fear as women did, but males display a more controlled reaction (Deng et al. 2016). In any case, female dogs, as a group, appeared to show more evident emotional responses both on the fear and happiness chemosignals and this result agrees with human studies reporting higher emotional reactivity in women (Gross and Levenson 1995; Brody and Hall 2000; Vigil 2009).

In conclusion, all reported differences indicated that coping strategies in emotional conditions could be sex dependent, at least in the breeds involved in our study. However, we are not able to disentangle whether such sex differences in the emotional expressivity are the results of a different emotional perception at sensorial level or a different emotional response. Future studies measuring physiological parameters could shed light on this point.
